# Best Practices for Partnering With Interpreters in Clinical Encounters: A Multimodal Approach for Teaching Medical Students

**DOI:** 10.7759/cureus.84948

**Published:** 2025-05-28

**Authors:** Oluwagbemisola Ibikunle, Olivia Kahn-Boesel, Sophie Yu, Stephanie Wang, Dorothy W Tolchin, Yarden S Fraiman, Jennifer Kasper, Wudeneh Mulugeta, Ann-Marie Thomas, Jaeyoon Cha, Salma Batool-Anwar, Shari Gold-Gomez, Christopher Kirwan, EJ Jarvie, Kate Wasden, Daniele Olveczky, Rose L Molina

**Affiliations:** 1 Anesthesiology, Northwestern University Feinberg School of Medicine, Chicago, USA; 2 Medicine, Massachusetts General Hospital, Boston, USA; 3 Medical Education, Harvard Medical School, Boston, USA; 4 Interpreter Services, Beth Israel Deaconess Medical Center, Boston, USA; 5 Physical Medicine and Rehabilitation, Massachusetts General Hospital, Harvard Medical School, Boston, USA; 6 Neonatology, Beth Israel Deaconess Medical Center, Harvard Medical School, Boston, USA; 7 Pediatrics, Brigham and Women's Hospital, Harvard Medical School, Boston, USA; 8 Medicine, Cambridge Health Alliance, Harvard Medical School, Cambridge, USA; 9 Physical Medicine and Rehabilitation, Massachusetts General Hospital, Boston, USA; 10 Physical Medicine and Rehabilitation, Spaulding Rehabilitation Hospital, Boston, USA; 11 Pulmonary, Critical Care, and Sleep Medicine, Brigham and Women's Hospital, Harvard Medical School, Boston, USA; 12 Interpreter Services, Massachusetts General Hospital, Boston, USA; 13 Medicine, Beth Israel Deaconess Medical Center, Harvard Medical School, Boston, USA; 14 Obstetrics and Gynecology, Beth Israel Deaconess Medical Center, Harvard Medical School, Boston, USA

**Keywords:** communication, interpreters, language, limited english proficiency, medical education, undergraduate medical education

## Abstract

Introduction: Effectively working with medical interpreters is a critical skill for all physicians. We partnered with interpreters, students, and faculty to co-design and co-teach an educational session to improve medical students' knowledge and confidence in working with interpreters.

Methods: The multimodal educational intervention lasted 120 minutes and included a didactic presentation, video role-play encounters, and a panel discussion with interpreters. The intervention was evaluated with pre- and post-session surveys. McNemar’s test was used to compare knowledge and confidence scores.

Results: A total of 161 pre-clinical medical students participated in the session. Students identified knowledge they learned, including the role of interpreters, the importance of not relying on family members as interpreters, the use of pre-encounter huddles, and appropriate physical positioning. After the intervention, the majority of students identified best practices for working with interpreters (n = 64 (57.7%), pre-session vs. n = 45 (71.4%), post-session; p = 0.04) and reported improved confidence working with interpreters (n = 73 (65.8%), pre-session vs. n = 60 (95.2%), post-session; p < 0.001).

Discussion: This innovative curriculum was unique in its interprofessional co-design and co-teaching approach and introduction of nuanced, contemporary clinical scenarios. Overall, this innovative curriculum was successful in enhancing student knowledge and confidence in working with interpreters.

## Introduction

Given the growing linguistic diversity in the United States, communicating effectively with interpreters is a critical skill for all physicians and imperative for equitable healthcare delivery. According to the U.S. Census Bureau’s 2021 American Community Survey, 67.2 million people speak a language other than English at home. Of these individuals, 25.5 million speak English less than “very well” according to the U.S. Census [1]. This population has been categorized as having “limited English proficiency,” which is defined as having difficulties reading, speaking, writing, and/or understanding the English language [2]. We prefer inclusive language and a strengths-based approach [3], so we will refer to this population as patients who speak languages other than English despite the inconsistency with how language proficiency is measured across studies. Patients who speak languages other than English are less likely to have access to healthcare and preventative services, demonstrate comprehension of and adherence to treatment plans, be satisfied with their care [4], and are more likely to have longer lengths of stay compared to English-speaking patients [5]. Effective communication between clinicians and patients who speak languages other than English is crucial for high-quality care and achieving health equity among this marginalized population.

With the growing number of individuals who speak languages other than English in the United States [6], skilled collaboration with qualified medical interpreters in clinical settings is increasingly important; however, ad hoc interpreters (e.g., staff or family members) or no interpreters are utilized in many situations [7], despite federal and other regulatory requirements for meaningful language access [8]. The use of ad hoc interpreters has been associated with lower patient satisfaction, poor medication adherence, and reduced understanding of medical conditions [9], while inclusion of certified medical interpreters in clinical encounters has been shown to reduce communication errors with clinically significant outcomes, including shorter lengths of stay [10,11]. In addition to their role in providing language interpretation, certified medical interpreters often serve critical roles in clarifying meaning and verifying understanding (e.g., informing the healthcare team about gaps in patient understanding and encouraging patients to ask questions) and in cultural brokering by serving as mediators between cultures and providing additional cultural context for mutual understanding [12].

There are many published curricula in graduate medical education (GME) (e.g., pediatrics, internal medicine, and emergency medicine) [13-15] and undergraduate medical education (UGME) that focus on teaching best practices of working with interpreters and patients who speak languages other than English [16-20]. However, with the emergence of artificial intelligence (AI) and the increased prevalence of virtual interpreters, few formal curricula address these contemporary clinical scenarios. Additionally, more health systems are beginning to implement qualified bilingual staff assessments to verify non-English language skills of healthcare workers [21,22], yet medical students are often left out of such assessment programs. We aimed to bridge this gap by adapting prior curricula targeted toward resident education [15] to the UGME setting and expanded to teach concepts of language justice [23,24] and cultural humility in complex settings, including working with patients who speak languages of lesser diffusion (i.e., indigenous languages), patients who are deaf/hard of hearing, virtual interpreters, and AI technology. We also included a section dedicated to qualified multilingual assessments, given the linguistic diversity of medical students and their experiences of being tasked inappropriately with interpreting for medical teams [25]. This curriculum was designed to help preclinical medical students develop a foundational knowledge base to address these nuanced scenarios prior to starting their clinical rotations. To expand on these crucial topics, our curriculum incorporates an iterative co-design [26] and co-teaching approach by faculty, senior medical students, and interpreters/cultural brokers, providing a range of perspectives to enhance the pedagogical approach with learners.

Building on prior literature [16-20], we co-designed a low-cost and easily reproducible session, inclusive of a didactic presentation, instructional role-play video discussion, and interdisciplinary panel discussion to teach best practices for working with interpreters during clinical encounters. We focused on equipping students with the knowledge and confidence to engage in both basic and complex interactions with interpreters effectively. The learning objectives of this curriculum were to define language justice as it applies in healthcare; list the roles of interpreters in healthcare teams for patients who speak languages other than English; identify common challenges and best practices for working with interpreters in clinical encounters; compare linguistic interpretation to mediation to cultural brokering; and identify the evolving role of virtual interpreters, machine translation, and AI in medical interpretation.

## Materials and methods

Education team

Our team consisted of eight physicians from six clinical fields (physical medicine & rehabilitation, obstetrics & gynecology, sleep/pulmonary medicine, neonatology, pediatrics, and internal medicine/primary care), five post-clinical medical students, three certified medical interpreters (Mandarin, Russian, and Haitian Creole), and one medical education administrative staff member. The interprofessional team was recruited from professional networks and a pre-existing working group dedicated to improving our institution’s longitudinal health equity and anti-racism curriculum. This curriculum was adapted from foundational work by Fune et al. [15], with expansion in content areas through a co-design approach, including biweekly to monthly meetings involving medical students, faculty, and certified medical interpreters across five major teaching hospitals. We aligned the session’s learning objectives with the competencies from the longitudinal health equity and anti-racism curriculum theme within the medical doctorate (MD) program by focusing on improving cultural responsiveness and preparing students to care for diverse patient populations (Liaison Committee on Medical Education Standard 7.6).

The initial version of this session was developed in spring 2023, and the revised versions were developed in winter 2023-2025 after incorporating student feedback. We present student data from the third iteration of the session targeted toward second-year preclinical medical students.

This study was deemed educational quality improvement by the Harvard Medical School Educational Scholarship Review Committee and was exempt from IRB review. The study was conducted in accordance with the Declaration of Helsinki.

Formative needs assessment

As a formative needs assessment for developing the didactic session, we distributed a voluntary survey to all 847 enrolled medical students. We received 68 responses, of which 36 students (53.0%) responded that they had not received any formal instruction in working with interpreters. Many students responded that they would like to have formal instruction on best practices for working with interpreters. We used data from this survey to identify gaps in student knowledge and designed a didactic presentation adapted from Fune et al. [15], with expansion on specific content areas to address these gaps. We also further explored the literature to identify published curricula about working with interpreters in clinical encounters [27,28], and we partnered with certified medical interpreters who have teaching expertise on this topic.

Educational curriculum

We presented a curriculum including a didactic presentation, video-recorded role-play discussion, and interdisciplinary panel discussion. Our curriculum was co-designed and also co-taught by physicians, senior medical students, interpreters, and cultural brokers.

The December 2024 version of the intervention was delivered as a 120-minute session, which included a 60-minute didactic presentation, a 30-minute segment of viewing a 15-minute video, and 15-minute discussion, and a 30-minute panel discussion with two interpreters. Two students and one faculty member led the didactic presentation, which was built on didactic slides from Fune et al. [15], with expansion to novel content as described.

We professionally recorded and edited two mock clinical encounters, one demonstrating areas in need of improvement and one demonstrating best practices. We developed a script focused on counseling about the human papillomavirus vaccine, so the students could witness a sensitive conversation with an interpreter. Two medical students role-played as the clinician, one bilingual Mandarin-speaking medical student served as the patient, and a certified Mandarin interpreter helped interpret. Following the didactic presentation, the first video was shown, followed by a group discussion to elicit thoughts about what could be improved. Next, the “best practices” scenario was played, followed by another large group discussion regarding differences observed between the two video scenarios. The sample script of the roleplay is presented in Appendix 1.

The third component of the session was an interdisciplinary panel discussion with two certified interpreters. We prepared some introductory discussion questions (Appendix 2) and used the remaining time to answer questions from the students. Students asked about the use of humor across language barriers, tips for performing a physical exam with an interpreter, breaking bad news, and the use of AI to translate written documents in the hospital. Panelists discussed the hospitals' policy regarding the use of AI for language access and ongoing pilot studies around the accuracy of AI translation for discharge instructions. This discussion was designed to be responsive to student-driven concerns and questions. The panel format could include a variety of individuals involved in caring for patients who speak languages other than English, such as certified medical interpreters, qualified bilingual healthcare providers, cultural brokers, and/or bilingual patients or family representatives.

Assessment

We developed a pre- and post-session survey (Appendices 3 and 4) to measure the students’ knowledge about the specific learning objectives. We refined the questions for clarity after pilot testing with previous cohorts of medical students. We administered the pre-session survey before the session and the post-session survey following the session. The pre-survey gathered student demographics, baseline knowledge, and what they hoped to learn from the session. The post-survey gathered students’ knowledge following the intervention, what they learned during the session, and any feedback. The pre-survey included 11 multiple-choice and two free-text questions that covered student demographics, baseline knowledge, and what they hoped to learn from the session. The post-survey included seven multiple-choice and three free-text questions about students’ knowledge following the intervention, what they learned during the session, and any additional feedback they had. We tabulated descriptive summary statistics of student responses to both surveys. We utilized McNemar's test to calculate p-values to determine statistically significant improvement in performance after completing the curriculum among students who completed both pre- and post-surveys. For the Likert scale question, McNemar's test was also used to determine statistical significance between pre-survey and post-survey results. “Somewhat confident” and “Very confident” were grouped into the “Confident working with an interpreter” category. “Not at all confident” and “Not very confident” were grouped into the “Not confident” category to produce a binary variable. We also analyzed free-text comments by coding them according to four major themes: how to work with interpreters, logistical support, cultural sensitivity, linguistic considerations, and support for the use of virtual and AI interpreters. We present pooled results from the December 2024 and March 2025 educational sessions.

## Results

Of the 161 enrolled students, 111 (68.9%) completed the pre-survey. Of these 111 students, 14 (12.6%) were born outside the US, 43 (38.7%) spoke a language other than English at home, and 22 (19.8%) had served as an interpreter for family or friends in a medical setting previously. Students described what they were looking forward to in the session, including “learning more about the dynamics between patient, provider, and bilingual family members of the patient,” “what to do when you know the interpreter is changing your wording,” and “what to say when a family member would like to interpret.”

**Figure 1:**
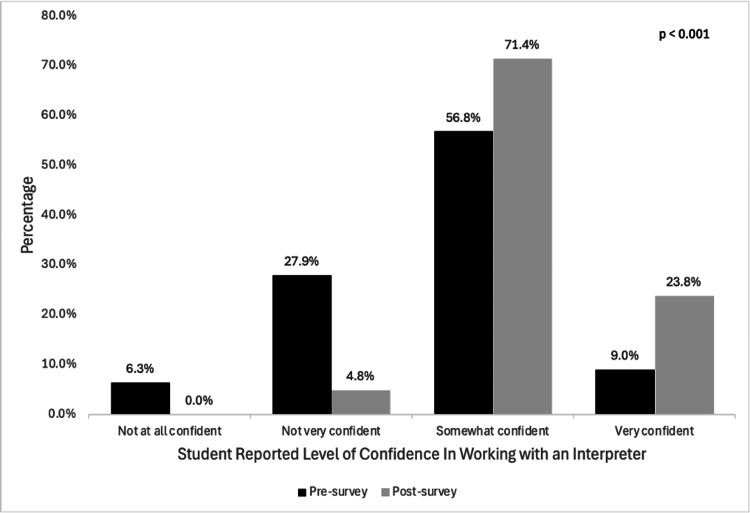
Students' self-reported confidence levels in working with an interpreter before and after the educational intervention (p < 0.001). McNemar's test statistic.

Sixty-three students completed the post-session survey. Quantitative results are summarized in Table 1. There was a statistically significant improvement in knowledge related to best practices for working with interpreters (n = 64 (57.7%) vs. n = 45 (71.4%), p = 0.04), documentation (n = 13 (11.7%) vs. n = 18 (28.6%), p = 0.03), virtual interpretation (n = 45 (40.5%) vs. n = 32 (50.8%), p = 0.04), and in confidence in working with an interpreter (n = 73 (65.8%) vs. n = 60 (95.2%), p < 0.001). For example, one student responded, “Learning about the positioning of the interpreter in the room to make the patient feel supported was new to me and very helpful; I also was very happy to learn about how to handle a situation where a family member offers to translate” (Table 2). Students' self-reported confidence levels before and after the intervention are shown in Figure 1. Student feedback included “The videos were very helpful” and “This was a wonderfully informative session.”

**Table 1:** Results of pre-session and post-session knowledge questions. McNemar's test statistic.

Survey question	Pre-session survey percentage correct, n (%)	Post-session survey percentage correct, n (%)	p-value
	N = 111	N = 63	
Which of the following describes the role(s) of a certified medical interpreter? Select all that apply.	82 (73.9%)	46 (73.0%)	0.60
Which of the following are best practices for working with interpreters? Select all that apply.	64 (57.7%)	45 (71.4%)	0.04
Which of the following is the minimal documentation required in the medical record?	13 (11.7%)	18 (28.6%)	0.03
How do cultural brokers and linguistic interpreters differ?	107 (96.4%)	60 (95.2%)	0.32
Please select all statements that are TRUE regarding AI interpreters.	57 (51.4%)	22 (34.9%)	0.07
Select all that apply regarding best practices for virtual interpretation.	45 (40.5%)	32 (50.8%)	0.04
Please describe your confidence in working with a certified medical interpreter for a patient encounter.	73 (65.8%)	60 (95.2%)	<0.001

**Table 2:** Illustrative themes and quotes from formative pre-session and post-session surveys of all iterations of the curriculum.

Theme	Pre-session survey: “What formal instruction would you like regarding working with interpreters?”	Post-session survey: “What are 1-3 things you learned in this session?”
How to work with interpreters	“How to introduce self and patient. How to be efficient.” “How to interact with interpreters, what to expect, general flow, dos and don’ts, what services interpreters provide versus what they don't.” “How to maintain flow and rapport”	“Address the patient, not the interpreter. Family members should not be relied upon for interpretation.” “It is good practice to do a pre-huddle and give the interpreter context before seeing the patient.” “How to prepare for a session with an interpreter; how to address the patient during a session.” “I really liked the point at the beginning about 'working with' vs 'using' an interpreter. I'm sure I've said 'using' an interpreter before, and I just thought that was a wonderful point about being intentional about the language we use for team members.”
Logistical support	“How to get an interpreter at that particular site, including phone/page, iPad, and what to do after hours, and how to handle need for translation of written materials (e.g., discharge summaries) since interpreters only do verbal communication.” “How to determine which language a patient needs an interpreter for, how to call an interpreter, which forms of interpretation are available (e.g., in-person, virtual, phone)”	“The positioning of the interpreter at the side of or behind the patient is empowering! I didn’t realize how accessible phone/Zoom interpreters were either.” “Working with interpreters in patient encounters should be documented with interpreter ID.” “Learned that you should be prepared to provide language accessible papers and handouts to patients prior to the visit.”
Cultural sensitivity	“Talking about pain in patients from cultures where this is not as openly discussed”	“During the physical exam, do not begin any maneuvers until the interpreter has finished translating and the patient has heard what the next step is.” “I learned that sometimes interpreters may interrupt to clarify or provide suggestions, which is helpful to know! I previously thought their only purpose was to provide direct translation.”
Linguistic considerations	“Learning how to troubleshoot describing things when there are not exact words to translate.” “Tips on different dialects (i.e., Moroccan Arabic versus other Arabic)”	“I also was very happy to learn about how to handle a situation where a family member offers to translate. It was very helpful to think and talk explicitly about the huddle.”
Support for the use of virtual and AI interpreters	“I hope to learn more about the different types of interpreters, best practices for virtual interpretation, and tips for AI use.” “How to work with an interpreter over the phone or iPad when an in-person interpreter is not available”	“I learned more about virtual interpretation.” “Always look at the patient!”

## Discussion

This multimodal curriculum was designed for the UGME setting with expansion to address complex scenarios in working with interpreters. Collaborating with an interdisciplinary working group, we co-developed and co-taught a class, including a didactic presentation, role-play video discussion, and interdisciplinary panel with interpreters. The co-design [28] and co-teach process was unique in its inclusive approach with students, interpreters, and faculty who developed the materials and led the sessions together. Students had the opportunity to learn directly from interpreters through a panel discussion, with whom they often do not have an opportunity to engage with during training, as well as from senior medical students, who shared their experiences about working with interpreters in clerkships. Furthermore, this curriculum addressed the concepts of working with interpreters within the framework of language justice [24].

This curriculum addressed best practices in regard to several specific patient populations, including those who speak languages of lesser diffusion and patients who are deaf/hard of hearing. Our session also covered contemporary scenarios for which best practices had not been systematically integrated into a formal UGME curriculum, including machine translation, AI and virtual interpreters, and qualified multilingual assessments for students and healthcare workers. In our review, these best practices have not been systematically integrated into a formal curriculum targeted for the UGME setting. With the rise of AI, machine-based translation services and applications are becoming increasingly relevant to clinical practice [29]. Our session was also novel in addressing appropriate use cases for these services in a clinical setting and provided students with practical recommendations to reduce the risk of error and promote success when interacting with interpreters in all settings.

Our multimodal curriculum enabled students to apply knowledge from the didactic presentation to identify areas for improvement in the first role-play video. These insights were reinforced through a second video showcasing best practices. Post-session surveys demonstrated that students gained new knowledge and highly valued the session. Students also reported increased confidence in working with interpreters and enhanced understanding of interpreter practices. The students appreciated the interdisciplinary approach and collaboration among faculty, students, and interpreters. The students also appreciated the discussion regarding the appropriate use of AI for language access in the context of current evidence, ethical frameworks, and hospital policies. We attribute part of the success of this session to the numerous improvements to this curriculum made since the initial version in spring 2023. We also acknowledge that the observed gains represent short-term outcomes, and long-term knowledge retention and behavior changes require additional assessment.

This educational intervention is limited by selection bias in the survey approach and the lack of a standardized patient encounter for students to apply new knowledge and receive formative feedback. This was not implemented due to the limited pool of standardized patients who are bilingual/multilingual, appropriate interpreters, and the financial costs of scaling to an entire class of students. We anticipate overcoming this limitation by implementing a standard clinical rubric for interpreter encounters during the obstetrics and gynecology and pediatrics clerkships, so preceptors can observe students in a clinical encounter and provide real-time feedback. We acknowledge that the relatively large proportion of medical students with multilingual backgrounds is a unique strength that may limit generalizability to other students who may have less exposure to languages other than English. We also acknowledge that non-response bias in the needs assessment may have influenced the initial curriculum development. However, we refined the curriculum based on student feedback over multiple years, leading to a more robust curriculum despite the initial low response rate to the needs assessment. While we did not have an external control group, we did have an internal control by surveying the same group of students before and after the educational curriculum. Another limitation to our curriculum is that only 63 of 161 students enrolled in the course completed the post-survey, which could lead to response bias. Furthermore, 43 (38.7%) students spoke a language other than English at home, which may not be representative of other medical student audiences. Finally, given that phone- and video-based interpreting are commonly used in clinical settings, especially post-COVID-19 pandemic, we could include a roleplay on how to use phone and virtual interpreter modalities in future iterations. Based on feedback from students, we have been progressively moving the session earlier in the preclinical curriculum. Our next session will be offered to a new cohort of first-year medical and dental students.

## Conclusions

Overall, this innovative curriculum with a didactic presentation, role-play-based video encounter, and interdisciplinary panel discussion with interpreters was successful in improving student knowledge and confidence in working with interpreters in clinical encounters. These concepts are foundational for all physicians and critical to achieving equitable care for the increasingly linguistically diverse patient population in the United States.

## Appendices

Appendix 1: Roleplay script

Background: A medical student is meeting a patient in a primary care clinic with the primary motive of discussing the human papillomavirus (HPV) vaccine. The student must recognize that the patient needs an interpreter, call for and briefly huddle with the interpreter, and work with the interpreter appropriately in the interaction. The first scenario does not go according to best practices. The second scenario proceeds according to best practices.

Length: Approximately five to seven minutes per scenario

Summary of Not-Best and Best Practices for Working With an Interpreter

**Table 3:** Best practices for working with an interpreter in video roleplay.

Areas for growth	Best practice
Not recognizing the need for an interpreter based on the medical record review	Recognize the need for an interpreter prior to encounter (proper chart review practices)
Asking family members to be interpreters	Only using certified medical interpreters employed by the hospital
No huddle with interpreter prior to the encounter	Huddle with the interpreter prior to the encounter
No acknowledgment of interpreter confidentiality	Allow interpreter to introduce themself and explain the confidentiality of the encounter
The interpreter is positioned between the patient and the clinician	The interpreter is positioned to the side of the patient
Clinician's eye contact with the interpreter	Clinician's eye contact with the patient
The clinician speaks to the interpreter, not the patient	Clinician speaks to the patient, not the interpreter
Speaking in “chunks” that are too long (or too short) to be interpreted	Speaking in appropriate short “chunks” (1-2 sentences), utilize teach back method
Using too much medical jargon	Using plain language
Printed materials are in English only	Printed materials are available in the patient’s preferred language
Rushing appointment due to interpreter use	Develop rapport throughout the encounter, including empathy and cultural humility, especially when language barriers are present

First Scenario: Areas for Growth in Working With an Interpreter

~Medical student knocks on the door and walks into the room. The patient is sitting in the room already~

Medical student: Hi there! My name is X, I am the medical student working with the team today. Are you Ms. Yu? (looks at clipboard, doesn’t make eye contact)

Patient: ~smiles, looks a bit confused~

Medical student: (talking quickly) So what brings you in today?

Patient (in broken English accent): Don’t speak English

Medical student: Oh, I didn’t know, I’m sorry! I can try to call an interpreter. I’ve never done it before, but I’m sure I can figure it out.

Patient (in broken English accent): My sister speak English. Can she come?

Medical student: Oh, perfect, we could ask her to interpret! Is she here?

Patient (in broken English accent): Oh, no….park..car

Medical student: No problem, I’ll find an interpreter.

(Student leaves, returns with interpreter, and directs interpreter to stand between student and patient)

Interpreter (in Mandarin): Hello, I’m Stephanie, the Mandarin interpreter.

Medical student: So, as I was saying earlier, what brings you in today?

-Interpreter interprets-

Patient: I am just here for a check-up. Also, a friend mentioned a vaccine to me, and I’m not sure I got it yet.

-Interpreter interprets-

Medical student: (looks directly at interpreter) Oh, great. Do you know which vaccine it is? I know there are a lot of vaccines out there, so it can be confusing to remember all of them. Also, some of them we need as adults and some we need as kids, so even sometimes I forget which ones we need and how many shots we need…

Interpreter (interrupts student given the length): I will interpret that now, if that’s okay, so I don’t forget

-interprets prior chunk for patient-

Patient: Thank you so much, so I was wondering if you could tell me about the cancer vaccine?

-Interpreter interprets-

Medical student (looks directly at interpreter): Hmm, you mean the human papillomavirus vaccine? It is a vaccine against the DNA virus that can cause cervical cancer!

-Interpreter interprets-

Patient: (looking a bit confused) That’s not what my friend said, but uh, I think so? Maybe?

-interprets-

Medical student: I can talk to you more about the vaccine. Do you know anything about it?

-Interpreter interprets-

Patient: I’m not sure, should I know something about it?

-Interpreter interprets-

Medical student (looks directly at interpreter): No, I just wanted to check! Do you know how HPV is transmitted? It is sexually transmitted, so the guidelines recommend that all young people get the vaccine as early as possible to get the full protection and benefits before starting to have sex.

-Interpreter interprets-

Patient: (looks uncomfortable/embarrassed/nervous) Uh, is this conversation private with the interpreter here? It feels like a bit of a sensitive topic to discuss.

-Interpreter interprets-

Medical student: Oh yes, definitely, everything is confidential! ~interpreter nods in agreement~

-Interpreter interprets-

Patient: Ok, thank you.

Medical student: So anyways, HPV is a virus that can be transmitted during skin to skin contact with someone who has the virus, usually in the setting of anal, vaginal, or oral intercourse.

-Interpreter interprets-

Patient: (looks embarrassed/uncomfortable) *nods*

Medical student: HPV is actually very common among sexually active adults, and it can lead to several kinds of cancer. The vaccine can help prevent those cancers. We have a handout on it if you want to read more about it...

-Interpreter interprets-

Patient: (looks at handout) - Oh, I’m afraid I don’t read English very well. Do you have this in Mandarin?

-Interpreter interprets-

Medical student: Oh, I’m sorry about that. We will have to get it translated! So, do you have any questions about the vaccine?

-Interpreter interprets-

Patient: I am not currently sexually active. Does that mean I don’t need the vaccine?

-Interpreter interprets-

Medical student: That’s a very good question. The vaccine is still recommended to prevent infection if you choose to become sexually active later. So, are you ready to get it? (Student looks rushed and ready to leave, given how long the appointment has taken already)

-Interpreter interprets-

Patient: (looking apprehensive) Uhhh, yes, I guess I will. I’m sorry that I had so many questions.

-Interpreter interprets-

Medical student: Oh, don’t worry. Also, there are three shots in total, so we will need to book 2 more appointments after this one.

-Interpreter interprets-

Patient: Oh, okay. It is sometimes difficult for me to get off work to go to the doctor, but hopefully I can make it work.

-Interpreter interprets-

Medical student: I am sure the front desk can work with you to find a good time to schedule your next appointment. It was nice to meet you!

-Interpreter interprets-

END

Second Scenario: Best Practices for Working With an Interpreter

The medical student reviews the patient’s chart and notes that her preferred language is Mandarin. The student calls to arrange an interpreter. The interpreter meets the student at the door. Introductions are exchanged, and the student briefly describes the encounter to the interpreter. The student knocks on the door and walks into the room with the interpreter. The patient is sitting in the room already.

Medical student: Hi there, are you Ms. Yu? (Pause to allow interpreter to translate)

-Interpreter interprets-

Patient: ~smiles, nods~

Medical student: My name is X, I am the medical student working with the team today. I have brought our amazing Mandarin interpreter, Stephanie, with me today.

(seats everyone so they can see each other and talk directly to the patient)

-Interpreter interprets-

Stephanie: ~Introduces herself with standard introduction, ensures that everything said during the encounter is confidential.~

Patient: Thank you for bringing the interpreter and for seeing me today. I was wondering, is it okay if my sister translates instead? She’s on her way now.

-Interpreter interprets-

Medical student: We are happy to have your sister join us. However, we usually prefer to have a medical interpreter who can help with the medical terminology and allow for your sister to be here as your support person, not your interpreter. Is that okay with you?

-Interpreter interprets-

Patient: Ok, that sounds good.

-Interpreter interprets-

Medical student: Do you mind telling me what brings you in today?

-Interpreter interprets-

Patient: I am just here for a check-up. Also, a friend mentioned a vaccine to me, and I’m not sure if I got it yet.

-Interpreter interprets-

Medical student: Got it. We will do a routine examination today. Do you happen to know which vaccine your friend was referring to?

-Interpreter interprets-

Patient: I am not sure, I think it may be a cancer vaccine?

-Interpreter interprets-

Medical student: Hmm, do you mean the HPV vaccine to prevent cervical cancer as well as other types of cancer?

-Interpreter interprets-

Patient: Yes, I think that’s the one!

-Interpreter interprets-

Medical student: Great, I can definitely talk to you more about the vaccine. First, I would like to get to know you a bit better.

-Interpreter interprets-

Medical student: To get a better idea of your understanding, can you tell me what you know about HPV?

-Interpreter interprets-

Patient: (looks uncomfortable) Oh, I don’t really know very much, I just heard from my friend that I should get it.

-Interpreter interprets-

Medical student: Got it, so let's start by talking about how HPV is transmitted. How does that sound?

-Interpreter interprets-

Patient: Ok.

-Interpreter interprets-

Medical student: HPV is a virus that can be transmitted during skin to skin contact with someone who has the virus, usually in the setting of sexual contact, like anal, vaginal, or oral intercourse.

-Interpreter interprets-

Patient: *nods*

Medical student: HPV is actually very common among sexually active adults, and it can lead to several kinds of cancer. The vaccine can help prevent those cancers, especially if the vaccine is given before someone starts to have sexual contact with others.

-Interpreter interprets-

Patient: Oh, got it. I am not currently sexually active. Does that mean I don’t need the vaccine?

-Interpreter interprets-

Medical student: That’s a very good question. The vaccine is still recommended to prevent HPV infection if you choose to become sexually active later.

-Interpreter interprets-

Patient: Oh, ok. I am worried about potential side effects. Can we talk about that?

-Interpreter interprets-

Medical student: Of course. The HPV vaccine is very safe and has many years of safety data. Possible side effects include pain and redness in the arm.

-Interpreter interprets-

Patient: *nods*

Medical student: Other side effects include fever, nausea, headache, and muscle/joint aches. These symptoms are rare, and if they occur, they usually resolve within a day or two.

-Interpreter interprets-

Patient: Got it, that all sounds fine. *still looks apprehensive*

-Interpreter interprets-

Medical student: You look a little concerned. What is on your mind right now?

-Interpreter interprets-

Patient: I just want to make sure that this vaccine won’t impact fertility or anything like that. I also don’t know if I really need it.

-Interpreter interprets-

Medical student: Got it, thank you for letting me know about your concern! The HPV vaccine isn’t associated with the risk of infertility. It is the only vaccine that has been shown to prevent cancer, so I recommend it to all my patients as early as they can possibly get it!

-Interpreter interprets-

Patient: Ok, I’ll get it today.

-Interpreter interprets-

Medical student: We can definitely give you the first shot today. There are three in total, so we will work on booking the other shots after this appointment. How does that sound?

-Interpreter interprets-

Patient: Sounds good to me.

-Interpreter interprets-

Medical student: Before we prepare for the first shot, do you mind summarizing what the shot is for and why it’s important so I can make sure we’re on the same page?

-Interpreter interprets-

Patient: So this is a three-part vaccine that protects against HPV, which is a virus that can be spread through sexual intercourse. This virus can cause different kinds of cancer, which can be prevented by the vaccine.

-Interpreter interprets-

Medical student: Perfect! I’ll let the nurse know that you’re ready for your first vaccine. Very nice to meet you today!

-Interpreter interprets-

Patient: Sounds good, you as well!

-Interpreter interprets-

END

Appendix 2: Panel discussion

We suggest allotting the last 30 minutes of the session time for this activity. Our panel consisted of two certified medical interpreters (Russian and Haitian Creole) employed by our affiliated hospitals. We recognize that the composition of your panel will depend on the availability of personnel at your institution. Ideal panel members should have experience working with patients who speak languages other than English and a strong commitment to serving this population.

Suggested panelists might include certified medical interpreters, qualified bilingual clinicians, cultural brokers (e.g., bilingual community health workers and patient navigators), and/or bilingual patients or family representatives.

In our session, a facilitator posed the following questions to the panelists (allotting around 15 minutes). Audience members were also given 15 minutes to ask additional questions. Below, we included several prompts to facilitate discussion (see list below). However, the panel can be structured based on the interests of your audience.

Starred (*) questions are based on questions published in Fune J, Chinchilla JP, Hoppe A, et al. Lost in Translation: An OSCE-Based Workshop for Helping Learners Navigate a Limited English Proficiency Patient Encounter. MedEdPORTAL J Teach Learn Resour. 17:11118. doi:10.15766/mep_2374-8265.11118 [15].

Panel Discussion Questions

Please introduce yourself and describe what experiences inspired you to choose your professional field.*

Please elaborate on the specific motivations or experiences that led you to become an interpreter.

Please describe the differences between cultural brokering or mediation and interpreting, and how this is relevant to your work.*

Please give some examples of cultural misunderstandings or barriers you’ve witnessed and the strategies you use to bridge cultural differences and ensure mutual understanding.

What challenges do you encounter in your work, and what strategies help you to overcome these challenges?*

These challenges can relate to specific cultural differences (e.g., alternative medicine, evil eye, traditional healers, etc.) or interactions with patients, other colleagues, or medical professionals.

Please describe a situation in which a medical error or unforeseen outcome occurred due to a language or cultural barrier.*

Detail the nature of the error and the contributing factors, such as a misunderstanding of cultural norms or the lack of an interpreter during the interaction.

What is the role (if any) for machine translation (e.g., Google Translate and other app-based services) or artificial intelligence in this space?

Different panelists may have different opinions on this point. Please support your opinion with relevant anecdotes if applicable.

What are the best practices or tips for doing the physical exam while working with an interpreter?

If a student volunteer is amendable, consider demonstrating a typical cranial nerve exam with feedback from the panelists regarding ways in which the interaction can be improved when using an interpreter.

What is one piece of advice you would like to impart on our learners today?*

Examples include tips and tricks or a lesson you learned from a challenge you encountered.

During our session, learners asked several questions that were addressed by our panelists and may be helpful to address during the session.

Is there a role for humor and jokes when communicating with a patient using an interpreter?

Different panelists may have different opinions on this point. Please support your opinion with relevant anecdotes, if applicable.

How do you approach a situation where the patient and interpreter are having a side conversation, and you feel that not everything is being interpreted for you (the clinician)?

Please describe personal experiences and key strategies to manage this interaction.

What are the best practices for giving bad news while using an interpreter?

Please describe personal experiences and key strategies to manage this interaction, relevant anecdotes may be helpful.

What does the availability of translated written documents look like in the hospital?

Please describe the availability and demand for these documents at your institution. Also, expand on the role of artificial intelligence in the development of these documents in the future.

What is your advice for managing patient concerns about the quality of medical interpretation?

Please describe the options for finding an alternative interpreter or filing a safety report.

Appendix 3: Pre-session survey

1. Please list the last four digits of your cell phone number: (four digits)

2. In what country were you born?

a. United States 

b. Other _________ (free text) 

3. What language do you speak at home?

a. English 

b. Other _________ (free text)

4. Have you ever been an interpreter in a medical setting (e.g., for family members or friends)?

a. Yes

b. No

5. Among the patients you have cared for during in-person encounters, how many of them have experienced language barriers?

a. None

b. 1-5

c. 6-10

d. 11-15

e. 15+

6. Which of the following describes the role(s) of a certified medical interpreter? Select all that apply.

a. Changing the meaning of what is said between the patient and the provider when necessary

b. Providing clarification to cultural concepts or words

c. Maintaining impartiality

d. Acknowledging interpreter confidentiality and patient privacy 

7. Which of the following are best practices for working with interpreters? Select all that apply.

a. Pre-encounter huddles 

b. Optimizing the physical positioning of the interpreter in the room

c. Allowing bilingual family members to interpret based on the patient's request

d. Speaking in short duration with plain language

e. Maintaining eye contact with the interpreter throughout the visit

8. Which of the following is the minimal documentation required in the medical record? Select all that apply.

a. Name of the interpreter

b. Language of interpretation

c. Interpreter ID number

d. Time of interpretation

e. Location of interpretation

9. How do cultural brokers and linguistic mediators differ?

a. Cultural brokers focus exclusively on language barriers, while linguistic interpreters address broader cultural issues.

b. Cultural brokers mediate cultural differences between patients and their healthcare teams, whereas linguistic interpreters primarily provide language access.

c. Cultural brokers only address specific cultural misunderstandings, while linguistic interpreters emphasize long-term relationship building.

d. Cultural brokers specialize in translation and interpretation, whereas linguistic interpreters navigate broader cultural nuances.

10. Please select all statements that are TRUE:

a. In the literature, AI interpreters have primarily been tested on written text.

b. There is no standardized validation of AI technology for oral communication in medicine.

c. AI interpreters require "post-editing" or confirmation of accuracy by a trained multilingual professional.

d. Harvard Medical School (HMS)-affiliated hospitals have policies that allow for AI interpreter use in healthcare.

11. Select all that apply regarding best practices for virtual interpretation:

a. Virtual interpretation is equivalent to in-person interpretation.

b. A virtual huddle should be avoided to preserve patient confidentiality and efficiency.

c. For virtual interpretation, position the device so that the patient has an unobstructed view of the interpreter.

d. For virtual interpretation, always keep the camera in the same place throughout the encounter.

12. Please describe your confidence in working with a certified medical interpreter for a patient encounter.

a. Very confident

b. Somewhat confident

c. Not very confident

d. Not at all confident

e. What do you hope to learn in this session? (Free text response)

Appendix 4: Post-session survey

1. Please list the last four digits of your cell phone number: (four digits)

2. Which of the following describes the role(s) of a certified medical interpreter? Select all that apply.

a. Changing the meaning of what is said between the patient and the provider when necessary

b. Providing clarification to cultural concepts or words

c. Maintaining impartiality

d. Acknowledging interpreter confidentiality and patient privacy

3. Which of the following are best practices for working with interpreters? Select all that apply.

a. Pre-encounter huddles

b. Optimizing the physical positioning of the interpreter in the room

c. Allowing bilingual family members to interpret based on the patient's request

d. Speaking in short duration with plain language

e. Maintaining eye contact with the interpreter throughout the visit

4. Which of the following is the minimal documentation required in the medical record? Select all that apply.

a. Name of the interpreter

b. Language of interpretation

c. Interpreter ID number

d. Time of interpretation

e. Location of interpretation

5. How do cultural brokers and linguistic mediators differ?

a. Cultural brokers focus exclusively on language barriers, while linguistic interpreters address broader cultural issues.

b. Cultural brokers mediate cultural differences between patients and their healthcare teams, whereas linguistic interpreters primarily provide language access.

c. Cultural brokers only address specific cultural misunderstandings, while linguistic interpreters emphasize long-term relationship building.

d. Cultural brokers specialize in translation and interpretation, whereas linguistic interpreters navigate broader cultural nuances.

6. Please select all statements that are TRUE:

a. In the literature, AI interpreters have primarily been tested on written text.

b. There is no standardized validation of AI technology for oral communication in medicine.

c. AI interpreters require "post-editing" or confirmation of accuracy by a trained multilingual professional.

d. HMS-affiliated hospitals have policies that allow for AI interpreter use in healthcare.

7. Select all that apply regarding best practices for virtual interpretation:

a. Virtual interpretation is equivalent to in-person interpretation.

b. A virtual huddle should be avoided to preserve patient confidentiality and efficiency.

c. For virtual interpretation, position the device so that the patient has an unobstructed view of the interpreter.

d. For virtual interpretation, always keep the camera in the same place throughout the encounter.

8. Please describe your confidence in working with a certified medical interpreter for a patient encounter.

a. Very confident

b. Somewhat confident

c. Not very confident

d. Not at all confident

9. What are one to three things you learned in this session? (Free text response)

10. Please provide any other comments about this session. (Free text response)
